# The Role of Snack Choices, Body Weight Stereotypes and Smoking Behavior in Assessing Risk Factors for Adolescent Overweight and Obesity

**DOI:** 10.3390/foods10030557

**Published:** 2021-03-08

**Authors:** Elena Raptou

**Affiliations:** Laboratory of Management and Marketing, Department of Agricultural Development, Democritus University of Thrace, 68200 Orestiada, Greece; elenra@agro.duth.gr

**Keywords:** snack choices, body weight stereotypes, smoking, BMI, obesity, endogenous treatment effects

## Abstract

This study investigated the relationship of behavioral factors, such as snack choices, obesity stereotypes and smoking with adolescents’ body weight. Individual-level data for 1254 Greek youths were selected via a formal questionnaire. Snack choices seem to be gender specific with girls showing a stronger preference for healthier snacks. Frequent consumption of high-calorie and more filling snacks was found to increase Body Mass Index (BMI) in both genders. Fruit/vegetable snacks were associated with lower body weight in females, whereas cereal/nut snacks had a negative influence in males’ BMI. The majority of participants expressed anti-fat attitudes and more boys than girls assigned positive attributes to lean peers. The endorsement of the thin-ideal was positively associated with the BMI of both adolescent boys and girls. This study also revealed that neglecting potential endogeneity issues can lead to biased estimates of smoking. Gender may be a crucial moderator of smoking–BMI relationships. Male smokers presented a higher obesity risk, whereas female smokers were more likely to be underweight. Nutrition professionals should pay attention to increase the acceptance of healthy snack options. Gender differences in the influence of weight stereotypes and smoking on BMI should be considered in order to enhance the efficacy of obesity prevention interventions.

## 1. Introduction

In recent years, the prevalence of obesity has dramatically increased to such a degree in developed countries that it can be characterized as an epidemic [1]. This major public health challenge is especially addressed among youth populations who may be affected by long-term complications, including maintaining excessive body weight into adulthood [2,3].

Over the past two decades, overweight and obesity rates in Greece have reached worrying levels [4] with scholars and mass media providing extended reports to communicate health and nutrition recommendations during this public health emergency. Large-scale surveys carried out in Greek youths have indicated a sharply increasing trend on overweight and obesity prevalence [5,6,7,8], while inter-country comparisons of adolescent obesity estimates have shown that Greeks present the highest overweight rates in the European Region reaching up to 33% [9]. Although the causal explanation of the rapid obesity acceleration in Greek adolescents is not quite clear [5], fast moving modernization and social changes have led to a lower adherence to the “traditional” Mediterranean diet and the adoption of a more western-type and energy dense diet [10,11,12]. In addition, the economic recession during the last decade was followed by severe austerity measures and material deprivation, which have altered the dietary habits of adolescents and their families leading to poorer nutrition choices [11]).

Given the complexity of the factors affecting adolescents’ body weight, interventions to combat overweight and obesity will likely be more effective if they address multiple contexts of influence, including eating patterns, behavioral characteristics, and lifestyle aspects. Snacking constitutes a key element of adolescents’ food consumption patterns and daily life because snacks are among the first product that youths buy with their own money outside their family environment [13,14] and have a crucial role in socializing [15]. Snacks between meals contribute approximately a quarter to the total daily calorie intake in adolescents [16,17], and there has been a noticeable upward trend in snacking over the past decades [18,19].

Although the consumption of snacks in addition to the main daily meals could be regarded as a contributor to overweight and obesity due to extra energy intake and increased eating frequency, a review of the recent literature provides ambiguous results [19,20,21]. Several studies have indicated that the frequency of snacking and the daily calorie intake of snacks have an inverse or a rather insignificant association with overweight and obesity risks [22,23]. However, these associations may be confounded by potential underreporting of snacking behavior that occurs more often in overweight than in normal weight respondents [23,24].

The majority of the literature has not provided conclusive findings on a potential causal relationship between snacking and obesity [22,25,26,27]. The associations among snacking, diet quality and body weight seem to be determined by the consumption patterns of the main meals and the choice of foods and beverages consumed at snack occasions [28,29]. For instance, frequent snacking may have a negligible impact on body weight and daily energy balance in case snacks replace main meals. On the other hand, frequent consumption of energy-dense and nutrient-poor snacks in addition to the main meals may lead to higher energy intake and subsequent weight gain [29]. In a recent study, Tripicchio et al. [30] showed that overweight youths consumed more snacks with higher calorie content at each snacking occasion compared with their normal weight counterparts. Furthermore, portion size, especially of energy-dense snacks, has substantially increased in the last decades resulting to a concurrent rise in energy intake [31,32] and subsequent overeating since more food is usually consumed when large portions are offered [33]. Therefore, the investigation of snacking habits and preferences in adolescents is of considerable interest among nutritionists and public health scholars in order to evaluate snack contribution to dietary intake, and proceed to effective interventions for the improvement of eating patterns and obesity prevention.

The interpretation of youths’ attitudes and perceptions toward body weight are also considered a cornerstone for increasing the efficacy of nutrition communication and promotion programs to adolescent populations [34,35]. Perceived stereotypes about body weight and body image may be correlated with adolescents’ actual body weight and may also affect their responsiveness toward health communication. Weight stigma usually encompasses stereotypes that overweight and obese individuals are lazy, less popular among siblings and unmotivated and lack in willpower and discipline [36,37,38].

Although there are societal beliefs supporting that obesity discrimination may offer some incentive for weight loss in overweight individuals [39], most of the literature has shown that weight stigma may lead to unhealthy behaviors. Experiencing weight-based discrimination has been associated with disordered eating patterns, extreme weight management practices, low self-esteem, depressive symptoms, social isolation, avoidance of physical activity, and increased weight gain over time [38,40,41,42,43]. Overweight adolescents usually have to confront stressful situations as incidental outcomes of weight stigma that may trigger the consumption of palatable and energy-dense foods [44,45]. Although recent studies have showed the effects of obesity stereotypes and discrimination on adolescents’ nutrition, health and social behavior, there still exists inadequate understanding of the potential impact of weight bias on adolescents’ body weight. Therefore, exploring weight stereotypes and their relationship with body weight should help improve adolescents’ dietary pattern and support behavioral strategies for weight loss, besides reducing weight discrimination and stigma.

Since obesity prevalence in Greek adolescents has reached alarming levels, there is an urgent need for adequately understanding their contributing factors and proceed to the implementation of effective nutrition and obesity prevention programs. Using cross-sectional data from a sample of adolescents in Greece, the present study sought to investigate various individual-level behavioral factors, such as snack consumption and body weight stereotypes and explore their influence on adolescents’ body mass index (BMI). Furthermore, this study assessed potential interactions between smoking behavior and body weight to further support previous evidence that multiple health risk behaviors usually co-occur [46]. For instance, overweight adolescents may be less risk-averse and adopt the smoking habit more easily than normal-weight peers.

Recent literature has provided conflicting results regarding the impact of smoking behavior on adolescents’ body weight. In particular, Jacobs [47] noted an inverse relationship between smoking and BMI, whereas Bertoni et al. [48] showed a higher prevalence of abdominal obesity in youth smokers than in nonsmokers. Lee et al. [49] stated an inverse relationship between body weight and daily smoking in girls, and also declared a negative association between perceived weight status and smoking rates. Boys with a BMI within the overweight to obese range had a lower likelihood of smoking. On the other hand, Lange et al. [50] encountered that a higher BMI predicted greater likelihood of regular smoking and increased cigarette consumption in female adolescents. Previous research has also noted that adolescents with weight concerns are susceptible to smoking behavior and more likely to smoke [51,52], whereas the perception that smoking can aid in weight management may constitute a crucial determinant of smoking initiation and the endorsement of habitual smoking behaviors [53,54,55]

## 2. Material and Methods

### 2.1. Data Selection and Sampling

The survey was designed and carried out in Greece, in the geographic divisions of Macedonia and Thrace, and focused on food attitudes and eating habits, lifestyle patterns, and body weight perceptions of adolescents 12–19 years old. This study was given ethical approval by the Ethics Committee of the Democritus University of Thrace (89/29/27). It lasted for ten months and was completed in October 2017. The whole procedure combined both qualitative and quantitative research methods. In the qualitative research implementation, data were obtained through a semi-structured group interview process, in which 40 adolescents were enrolled in six focus groups discussions. The qualitative research analysis offered useful insights into adolescents’ eating behavior, lifestyle factors, body weight perceptions, and stereotypes.

The main findings of the qualitative research were used to construct part of the formal questionnaire employed in the subsequent quantitative research. In particular, adolescents’ responses and comments on snacking choices/preferences and perceptions toward body weight and body size were used to construct the multi-item scales describing snacking behavior and body weight stereotypes. The quantitative research (formal) questionnaire mainly included closed-ended questions to depict adolescents’ snacking choices, body weight stereotypes, smoking behavior, and sociodemographic characteristics, such as gender and parental educational level. Only four open-ended questions were adapted to state adolescents’ age, height, body weight, and household size. The questionnaire was pretested on a small group of 50 adolescents for evaluating its comprehension and acceptability and its length and adherence.

Individual-level data were collected via the formal questionnaire that was administered to adolescents 12–19 years old. Participation was voluntary and the average time for the questionnaire completion was 10–15 min. A passive parental consent procedure was employed; under which parents were provided with adequate information about the study purposes and parental no response was regarded as permission for adolescent’s participation. Previous research has noted that passive parental consent is linked with higher response rates, whereas employing active consent procedures may exclude specific socio-demographic and high-risk groups [56,57,58]. In particular, adolescents with non-consenting parents have been found to be more involved in risk behaviors (e.g., cigarette smoking, alcohol drinking, and drug usage) [58,59,60] and they are more likely to have a higher body weight [57,61].

The study area consisted of all the 16 regional units of the selected study area, grouped into three administrative regions (Central Macedonia, Western Macedonia, and Eastern Macedonia and Thrace). The sample size was calculated according to the total size of adolescents’ population in the selected area for a 2–3% margin of error using a 95% confidence interval [62]. The total calculated sample size was divided among the regional units using a simple percentage. Participants were recruited through cultural activities, youth centers, sport clubs and recreational settings (e.g., music festivals, clubs, restaurants, and café). The data were selected outside of the school system to include adolescents with chronic absenteeism and youths who had dropped out of school [62]. The response rate for this study was 88% resulting to the selection of 1254 completed questionnaires.

### 2.2. Measures

Adolescents reported their height and weight and Body Mass Index (BMI) was calculated as body weight in kilograms divided by the squared height in meters. BMI assessed through self-reported weight and height has been found to be a valid measure of body weight among preadolescents and adolescents [63,64]. Participants were classified according to the IOTF (International Obesity Task Force) extended BMI cut-off points for thinness, overweight and obesity [65]. A four-point ordinal indicator was constructed taking the value of 1 for underweight adolescents, the value of 2 for normal weight and the values of 3 and 4 for overweight and obese respondents, respectively.

Adolescents were asked if they had smoked in the past 30 days before completing the formal questionnaire. Those who reported that they had smoked at least one cigarette in more than one day over the past 30 days were considered as current smokers [66]. Smoking dichotomous indicator took the value of 1 for current smokers and 0 for non-smokers.

Furthermore, snacking behavior was explored after adopting a conservative approach to describe snacking as any food consumption outside the three culturally accepted “main” meals [18,20]. In particular, all food items, which were consumed outside of breakfast, lunch, and dinner (>30 min) were included in snack foods [67,68]. The introductory question was “How often do you choose the following snack types?”. Snack choices were defined through a multi-item scale including seven indicators that were employed to measure consumption frequencies of specific food groups. The construction of the scale was based on the main findings from the preceding qualitative research design. These food groups included dairy products (milk, plain yogurt, yogurt desserts, cheese, etc), savory foods (sandwiches, pies, pizzas, and burgers), deep fried foods (chips, and crisps), confectionery (chocolates, biscuits, cakes, cream pie, apple pie, croissants, etc.), sugar sweetened beverages (carbonated drinks, and juices with added sugar), cereals/nuts (including cereal bars), and fresh fruits and vegetables. All the items were scored on a seven-point frequency scale ranging from several times per day to never (several times per day, daily, 4–6 times/week, 1–3 times/week, 1–3 times/month, less than once a month, never). The five categories of the scale were collapsed into two categories with a frequency of an at least “daily” to be the cut-off point to indicate frequent consumption.

To elicit adolescents’ attitudes toward weight and body size, participants were asked to score an eight-item variable on a five-point Likert scale ranging from “totally disagree” to “totally agree”. The items for this scale were drawn from the focus groups discussions in the primary qualitative research design. A factor analysis through principal component analysis (PCA) with Varimax rotation was employed to model the observed variables in terms of a smaller number of underlying unobservable factors. The factor analysis provided a two-component solution and the total variance explained was 66.293%. To assess the reliability of both components, the Cronbach’s alpha coefficients were evaluated indicating that the internal consistency of each factor is satisfactory. Table 1 analytically presents the main findings from the factor analysis and the reliability analysis application. To create measures of weight stereotypes, the detached items of each factor were added and the sums were dichotomized in order to construct two indicators for delineating endorsement of thinness and anti-fat attitudes, respectively.

The standard covariates pertained to socio-demographic characteristics, such as gender (binary variable, 1 for girls, 0 for boys), age (continuous variable), household size (continuous variable), and parental educational attainment. Two separate indicators were constructed to distinguish between maternal and paternal education (1 for higher educational attainment, 0 for a lower educational level).

### 2.3. Empirical Framework

This study explored the influence of snack consumption, body weight stereotypes and smoking behavior on adolescents’ BMI. The relationship between smoking and body weight is complex and potential endogeneity may lead to biased estimates. Endogeneity is attributed to situations in which an explanatory variable correlates with the disturbance term of the regression equation [69]. In case endogeneity is ignored, the obtained estimates may undermine the validity of the findings. Sande and Ghosh [70] provide an extensive discussion on endogeneity in survey research. Endogeneity may arise due to (i) reverse causality (simultaneity), (ii) omitted variables, and/or (iii) measurement errors [69,70].

In case of reverse causality, two variables are codetermined and each one affects the other. According to previous research, there is evidence of reciprocal relations between smoking behavior and body weight, with smoking behavior affecting weight and weight status influencing smoking habits [55]. If an endogenous variable (i.e., smoking indicator) is treated as exogenous in regression equation, then endogeneity issues may result in biased estimations [70].

Omitted variable bias may be attributed to uncontrolled confounding variables. In that case, an unobserved variable is correlated with both an independent variable in the model and with the error term. Therefore, unobserved characteristics may influence smoking behavior and body weight simultaneously. For instance, emotional states, such as depression and stress may be related with both smoking [71,72] and body weight [73,74].

Measurement errors may also incite endogeneity because of two reasons: (i) measurement errors in the independent variables (e.g., smoking behavior) resulting in attenuation bias [69,70], and (ii) measurement errors attributed to common method bias. In our case, smoking status and adolescents’ weight and height were self-reported. Factors associated with social desirability and social norms could affect self-reporting on those measures.

To obtain consistent estimates in the presence of potential endogeneity issues, our empirical analysis was based on the endogenous treatment effects model [69]. Since our outcome of interest was adolescent’s body weight expressed as an ordinal indicator, we adopted the approach introduced by Gregory [75]. More specifically, in the endogenous treatment effects model with an ordered outcome the dependent variable was specified through BMI classification and measured on an ordinal scale ranging from 1 to *J* (*j* = 1,…,*J*). Thus, the BMI equation (outcome) can be expressed as [75,76,77].

(1) $$Z_{i}=\;\left\{\begin{array}{c} 1\;if\;\;\;-\infty <Z_{i}^{*}<\mu_{1}\\ 2\;\;\;if\;\;\mu_{1}\leq Z_{i}^{*}<\mu_{2}\\ .\\ .\\ .\\ J\;if\;\mu_{j}\leq Z_{i}^{*}<\infty \end{array}\right\}$$

where *μ_j_*’s refer to threshold parameters and $Z_{i}^{*}$ is the latent outcome variable for the *i*th adolescent defined as

(2) $$Z_{i}^{*}=X_{i}\beta_{1}+Y_{i}\beta_{2}+\varepsilon_{i}$$

where $\varepsilon_{i}$ is the error term, $X_{i}$ is a vector of the explanatory variables including participant’s gender, age, household size, maternal and paternal educational attainment, snacking behavior, and weight stereotypes (endorsement of thinness, and anti-fat attitudes), and $Y_{i}$ describes respondent’s smoking behavior. In particular, $Y_{i}$ ∈ {0,1} corresponds to the binary treatment variable that takes the value of one for adolescents who reported current smoking and zero for non-smokers. The treatment equation can be expressed as

(3) $$Y_{i}=\left\{\begin{array}{c} 1\;if\;X_{i}\gamma_{1}+W_{i}\gamma_{2}+u_{i}>0\\ \;0\;if\;X_{i}\gamma_{1}+W_{i}\gamma_{2}+u_{i}\leq 0 \end{array}\right\}$$

where $u_{i}$ is the error term and $W_{i}$ is an exogenous instrumental variable describing exposure to maternal smoking (taking the value of 1 for adolescents whose mother was classified as current smoker and zero otherwise). In accordance with recent research, exposure to maternal smoking comprises a significant risk factor for youth’s smoking behavior [78,79,80], but it is unlikely to have a direct impact on BMI.

The error terms *ε_i_* and *u_i_* are assumed to follow a bivariate normal distribution with mean zero and covariance matrix of

(4) $$\left[\begin{array}{cc} \sigma^{2} & \rho \sigma \\ \rho \sigma & 1 \end{array}\right]$$

where *ρ* assesses the correlation between the treatment errors and the outcome errors. We also assumed that both ***X_i_*** and *W*_i_ are exogenous and unrelated to the error terms. Finally, Equations (1)–(3) were fitted via maximum likelihood [75,76,81].

To further evaluate the endogenous treatment effects in analyzing the association between smoking behavior and body weight, the marginal treatment effects (MTE) are also estimated [75,81]. The MTE indicate the average effect of smoking on the BMI of adolescents who are on the margin of indifference between being a current smoker and abstaining from smoking [76]. To enhance the reliability of our findings, the results of the univariate ordered probit model were also exhibited, in which potential endogeneity problems were ignored [82]. All analyses were conducted separately for boys and girls to explore potential differences in BMI determinants between genders.

## 3. Results

Sample description is analytically presented in Table 2. The majority of participants (65.9%) were classified as normal weight, 29.9% were overweight and obese, and only 4.2% were ranked as underweight. In our sample, 29% of adolescents were current smokers, with 63% of smokers being males. The Pearson Chi-Square test explored statistically significant differences in BMI, snack choices, body weight stereotypes, and smoking behavior between boys and girls and resulted in statistically significant associations of gender with most of the aforementioned indicators. More girls than boys had a normal body weight (74.2% vs. 57.3%), whereas the percentages of overweight and obese were increased in males. Furthermore, substantially higher percentages of girls than boys showed a clear preference for healthier snack options with a higher consumption frequency of dairy products (78.7% vs. 70.7%), fresh fruits and vegetables (48.2% vs. 26.1%), and a less frequent consumption of more energy dense foods, such as deep fried foods (4.1% vs. 17.6%), confectionery (12.3% vs. 29.5%), sugar sweetened beverages (23.9% vs. 36.2%), and sandwiches/pies/pizzas (25.4% vs. 46.7%). With respect to weight stereotypes, a notable proportion of respondents of both genders expressed anti-fat attitudes (60.8%), whereas adolescent boys were more likely to endorse the thin body stature compared with girls (43.5% vs. 35.7%). Non-parametric tests also showed that boys were more susceptible to smoking behavior than girls (36.7% vs. 20.9%) (Table 2).

Table 3 presents the univariate ordered probit model estimates, in which there is no assumption of endogeneity issues. The smoking indicator was found to be statistically significant with a negative sign in the regressions for both boys (*β* = −0.275, *p* < 0.10) and girls (*β* = −0.555, *p* < 0.01), implying that adolescents smokers are more likely to have a lower BMI compared with nonsmokers.

The results of the estimation of the endogenous treatment effects model are reported in Table 4 and Table 5. The instrumental variable employed for the analysis procedure in the treatment equation was statistically significant with a strong predictive power (Table 4). As expected, exposure to maternal smoking had a positive influence on adolescents’ smoking prevalence in both genders (full sample: *γ* = 2.274, *p* < 0.01, boys: *γ* = 2.367, *p* < 0.01, girls: *γ* = 2.336, *p* < 0.01). Body weight stereotypes and snack choices were also associated with smoking behavior. In particular, boys susceptible to the thin ideal had a lower probability of smoking (*γ* = −0.914, *p* < 0.01), whereas girls’ smoking behavior was inversely related with anti-fat attitudes (*γ* = −0.347, *p* < 0.10). Concerning snack behavior, frequent consumption of fatty snacks, such as confectionery and savory snacks (e.g., pies, and pizza), was positively related with current smoking in both genders. Contrary to our expectation, more frequent consumption of healthier snack options, such as dairy products and fresh fruits and vegetables increased the probability of smoking in adolescent girls (Table 4).

Table 5 summarizes the outcome equation (BMI) estimates of the endogenous treatment effects model procedure. The estimated correlation coefficient between the treatment errors and the outcome errors was statistically significant in the male subsample regression (*ρ* = −0.624, *p* < 0.01), implying that unobservables that influence smoking behavior tend to move with unobservables related with body weight. Therefore neglecting potential endogeneity may result to biased estimates of smoking coefficient that will not present the true effect of smoking behavior on adolescents’ body weight.

Snack choices were found to influence adolescents’ BMI (Table 5). In particular, the propensity for a higher BMI was augmented in adolescent male consumers of dairy products (*β* = 0.207, *p* < 0.10) and deep fried foods (*β* = 0.669, *p* < 0.01). Frequent consumption of confectionery foods and sugar sweetened beverages also increased the probability of overweight and obesity in both boys (*β* = 1.128, *p* < 0.01; *β* = 0.246, *p* < 0.05) and girls (*β* = 1.796, *p* < 0.01; *β* = 0.509, *p* < 0.01). Furthermore, energy dense snack options, such as sandwiches, pies, and pizzas enhanced weight gain in both genders. On the other hand, young males with a stronger preference for cereals/nuts had a lower likelihood of body weight increase (*β* = −0.857, *p* < 0.01). Frequent fruit and vegetables consumption had a contradictory influence on BMI between genders. Thus, snack preferences for fruits and vegetables were inversely associated with girls’ BMI (*β* = −0.094, *p* < 0.05), whereas boys who opted for fruits and vegetables had a higher likelihood of weight increase (*β* = 0.104, *p* < 0.05).

Adolescents’ susceptibility to body weight stereotypes was found to be a strong predictor of BMI in both genders (Table 5). The endorsement of thinness was positively related with the BMI of both adolescent boys (*β* = 0.436, *p* < 0.01) and girls (*β* = 0.255, *p* < 0.05). In contrast, anti-fat attitudes had a negative link with the BMI of male adolescents (*β* = −0.377, *p* < 0.01), although their influence on females’ body weight was negligible.

In comparison with the univariate ordered probit model results (Table 3), smoking estimates in the outcome equation were different in size and sign, especially in male youths (Table 5). Male smokers were more likely to have a higher BMI (*β* = 0.369, *p* < 0.05), whereas smoking behavior was negatively associated with girls’ body weight (*β* = −0.469, *p* < 0.05). Therefore neglecting potential endogeneity may result in biased estimates of smoking coefficient that will not reflect the true effect of smoking behavior on adolescents’ body weight. The marginal effects for both the endogenous treatment effects model and the univariate ordered probit model are provided in Table 6. In comparison with the univariate ordered probit marginal effects for smoking indicator, the marginal treatment effects differed in size and sign. The estimated marginal treatment effects for male adolescents indicated that cigarette smoking decreases the probability of being underweight by 1.8%, while increases the probability of being obese by 4.4%. On the other hand, smoking influence had an opposite direction in girls’ body weight with female smokers presenting a higher likelihood of underweight by 6% and a lower likelihood of being obese by 2.2%.

## 4. Discussion

In line with previous work, this study indicated significant differences in snacking behavior between adolescent males and females [14,83]. Although both healthy and unhealthy snack types were reported by participants, consumption patterns seem to be gender specific. In particular, more girls than boys opted for healthier snack types, such as fresh fruits and vegetables, whereas male adolescents showed a stronger preference for less healthful snack options, such as deep fried foods, sugar-sweetened beverages, and confectionery and savory snacks. Therefore, male adolescents seem to prefer the more filling, baked snacks, while their female counterparts may choose less filling types [14].

Estimations of the endogenous treatment effects model indicated that energy dense snack choices (confectionery, sugar-sweetened beverages, and savory snacks) augment adolescents’ ΒΜΙ. Young boys who regularly consumed deep fried snacks were also more likely to be overweight. On the other hand, healthier snack options, such as cereal and nut snacks had an inverse effect on body weight of both genders, whereas frequent consumption of fresh fruits and vegetables decreased girls’ BMI. Contrary to our expectation, a daily consumption of fruit and vegetable snacks was positively associated with BMI in adolescent males. A possible explanation can be the snack intake patterns of boys that mostly included energy dense foods (e.g., sugar sweetened beverages, and savory foods). Therefore, youth males who reported frequent fresh and fruits consumption at snack occasions may also consume foods from other snack categories on a daily basis. In that case, the intake of fruits and vegetables does not counteract the intake of higher-calorie snacks, but it is added to the total energy intake, leading to a positive energy balance and a subsequent weight gain.

Recent evidence suggested an unclear relationship between snacking frequency and weight gain [26,27,29,32]. In a thorough review, Njike et al. [19] noted that this discrepancy may be attributed to various factors including the different types of snack foods (e.g., energy dense or nutrient dense types), the outcome measures (e.g., BMI in comparison with waist circumference), and the differences in study populations and methodological approaches. Furthermore, potential underreporting of snacking behavior, which occurs more often in overweight individuals [24,84] in conjunction with the use of different definitions of snacks in the literature may provide equivocal conclusions about the relationship between snacking and obesity [85].

Although there is a long-held concern that snacking in addition to main meals may increase the probability of obesity development [26,86,87], it seems that snacking per se is not negatively associated with dietary quality. Eating snacks between the three main meals may help adolescents to fulfill their high energy and nutrition requirements, especially during puberty and provide a wider variety in food choice [32]. According to Hartmann et al. [88], frequent snacking may be linked with both healthy and unhealthy dietary patterns and hence, the relationship between snack choice and body weight seems to be principally defined by the consumption patterns of the main meals and the food types that are consumed at snack occasions [28,29,30]. In case snacks are consumed in place of meals, frequent snacking may have a negligible effect on body weight. Recent literature also showed that the consumption of high-fiber and/or high-protein snacks has no significant effect on body weight, whereas high-fat and high-sugar snacks may be associated with weight gain [19]. Consequently, nutrition and health interventions should focus on orientating youths toward the adoption of healthy snack habits in order to enhance nutrition knowledge and skills and decrease obesity risks. Previous studies have indicated that adolescents mostly rely on prior information and knowledge since they comprise the main criteria by which they judge the healthiness of snacks, whereas food label usage is limited [32,89]. Vila-Lopez et al. [90] also added that youths usually prioritize visual cues (i.e., size, images, colors, etc.) than informational cues (labels) in the packaging of low-fat and healthy foods. Food managers should pay attention to visual elements in order to attract young individuals’ attention and encourage healthy eating behaviors. Furthermore, social factors associated with peer influence play a major role in dietary behavior since snack foods are often chosen and consumed into peer groups [91].

Our findings also showed that the great majority of adolescents were susceptible to body weight bias and were mostly influenced by anti-fat attitudes. The endorsement of thinness was positively associated with BMI in both genders, indicating that adolescents with a higher body weight are more likely to express explicit positive attitudes toward the thin body stature and assign positive attributes to lean peers. On the other hand, anti-fat biases were inversely correlated with male adolescents’ weight status, showing that overweight boys are less likely to endorse stigmatizing beliefs toward obesity. A recent study also illustrated differences in perceptions about overweight and obesity among different BMI categories [35] with normal weight adolescents linking obesity with laziness and their overweight counterparts supporting that obesity is mostly attributed to heredity. Youths’ attitudes toward body weight and obesity seem to be influenced by public and media perceptions, which often generalize about obese individuals as those who lack discipline in eating behavior and controllability [35].

Perceptions of “right” body size are mostly affected by cultural ideals of physical attractiveness and are usually reinforced by adolescents’ immediate environments (i.e., family, peers), television and social media [34,92]. Even in children’s television shows, overweight characters are usually presented as being aggressive, unhealthy, and unpopular, whereas characters with slim figures are presented as being popular, polite, and attractive [38]. In another study conducted in adolescent populations, Eisenberg et al. [93] indicated that weight-stigmatizing incidents predominated in popular adolescent television programming and the prevalence of these incidents was much higher for youth-targeted than general audience-targeted shows. Thus, adolescents over-exposed to weight stigmatizing stimuli may become more responsive to contextual factors condoning discriminatory anti-fat views and internalize weight stereotypes. The adoption of negative attitudes toward obese peers could escalate weight stigma and intensify stress and depressive symptoms on overweight and obese adolescents.

Concerning the relationship between smoking behavior and body weight, our empirical findings revealed that ignoring potential endogeneity issues can lead to biased estimates of smoking on BMI and also indicated gender differences in the association of smoking with BMI. In particular, male smokers had a significantly higher probability of being obese than their nonsmoking counterparts. On the other hand, similar to Lee et al. [49], youth female smokers were more likely to be underweight and less likely to be obese compared to nonsmoking girls.

These findings underline the role of gender as a moderator of the relationship between body weight and smoking and further support previous studies highlighting the role of smoking as a perceived weight control strategy, especially in girls [49,50,53,54,55]. It is possible that female respondents with a lower BMI use cigarette smoking as a weight maintenance tool since recent evidence has also indicated that girls are more susceptible to weight issues and less satisfied with their weight than their male counterparts [49,50]. Furthermore, smokers seem to be more concerned with their appearance compared to nonsmokers [94], leading to increased probabilities for female smokers to link smoking behavior with weight control.

The positive relationship between smoking and BMI in boys may also suggest weight loss efforts using smoking as a means of BMI decrease. In addition, our analysis has also confirmed that poor snacking habits have a positive influence on smoking behavior and male smokers opt for less healthy snacks, such as confectionery and savory (e.g., pizzas, and pies). Therefore, the positive association between smoking status and body weight may be attributed to unhealthy eating patterns, such as frequent consumption of energy dense snacks, which seem to comprise a significant predictor of both BMI and smoking status in male youths. Furthermore, it is plausible that there may be a mutual relationship between different health-risk behaviors declaring a higher likelihood of smoking in overweight adolescent compared to normal-weight peers [46].

Our findings further emphasized the relationship between smoking and body weight and also revealed a crucial male-female difference. It seems that in young underweight girls, the desire for the thin body ideal may provide disincentives for smoking cessation and hinder compliance to antismoking regulations. On the other hand, the positive influence of smoking on obese males challenges the widespread belief that smokers have a lower body weight implying that health policy interventions could be oriented to battle both smoking and obesity simultaneously.

The main limitations of this study derive from the cross-sectional nature of the data employed that does not allow inferring causal relationships. Longitudinal follow-up may provide additional information on the impact of the key variables on adolescents’ body weight. Second, the study participants were recruited from northern Greece, so there may be uncertainty about the relevance and the applicability of the findings to the rest of the country. Regional differences in culinary culture may have an effect on eating patterns and preferences, which can influence the adolescents’ BMI distribution across Greece. Finally, the usage of self-reported measures may lead to over- or under- reporting. Although several studies have indicated that BMI calculated from self-reported height and body weight is a reliable weight measure for predicting obesity [63,95], other studies have shown an overestimation of self-reported body height and an underestimation of weight parameters [96,97]. Self-reported smoking behavior could also be biased since some adolescents may be reluctant or unwilling to reveal their smoking habits [49]. In addition, snack choices may be affected by potential underreporting, which seems to take place more often in overweight than in normal weight respondents [24,84].

## 5. Conclusions

In conclusion, snack preferences seem to be gender specific with girls consuming healthier snack types (fresh fruits, and vegetables) and boys opting for high-calorie snack options (deep fried foods, sugar-sweetened beverages, confectionery, and savory snacks). In addition, our findings further support previous evidence that the relationship between snacking and weight status can be determined by the choice of food types consumed at snack occasions. Thus, frequent consumption of energy dense snacks was found to increase BMI in both genders, whereas nutrient dense snack types had an inverse association with body weight of adolescent girls and boys. The great majority of adolescents also seem to be susceptible to weight bias. Adolescents with a higher body weight were more likely to assign positive attitudes to lean peers and overweight boys were also less likely to endorse stigmatizing beliefs toward obesity. Since perceptions of “right” and “ideal” body size are mainly attributed to cultural models of physical attractiveness, nutritionists and health marketers should consider differences in weight attitudes between genders and among different weight groups in order to promote healthy dietary behaviors with greater efficacy. This study also underlined the role of gender as a moderator of the association between body weight and smoking behavior. Male smokers were more likely to be obese, whereas youth female smokers had a higher probability of being underweight and a lower probability of being obese compared to nonsmoking counterparts. Thus, the higher BMI in male smokers may be attributed to less healthy food choices. On the other hand, adolescent girls seem to use smoking for weight management or as a diet strategy. Nutrition education and policy should take into consideration adolescents’ vulnerability and identify the special needs of different segments in order to design effective nutrition and health interventions.

## Figures and Tables

**Table 1:** Factor analysis results on body weight stereotypes.

Variables	Loading	Factor	Cronbach α
Lean people are more attractive	0.964	Endorsement of the thin body stature	0.914
Lean people are more successful	0.955		
Lean adolescents are more popular at school	0.647		
Lean people are more self-confident	0.963		
Overweight people are more lazy	0.647	Anti-fat attitudes	0.667
Overweight people have less friends	0.679		
Overweight people have only themselves to blame for their weight	0.732		
Overweight people are less happy	0.745		

**Table 2:** Sample characteristics (adolescents 12–19 years old).

Variables	Total (N = 1254)	Boys (N = 621)	Girls (N = 633)	Statistical Tests	
				*t*-Test	*p*-Value
Age ^1^	14.937 (1.529)	15.137 (1.604)	14.741 (1.426)	**4.622**	**0.000**
Household size ^1^	4.131 (0.935)	4.130 (0.982)	4.131 (0.886)	−0.013	0.990
				**Pearson** **Chi-square**	***p*-value**
Higher maternal educational level	384 (30.6%)	168 (27.1%)	216 (34.1%)	**7.375**	**0.007**
Higher paternal educational level	430 (34.3%)	237 (38.2%)	193 (30.5%)	**8.194**	**0.004**
**Snack choices**					
Dairy products	437 (74.7%)	439 (70.7%)	498 (78.7%)	**10.570**	**0.001**
Deep fried foods	135 (10.8%)	109 (17.6%)	26 (4.1%)	**60.164**	**0.000**
Confectionery	261 (20.8%)	183 (29.5%)	78 (12.3%)	**55.917**	**0.000**
Sugar sweetened beverages	376 (30.0%)	225 (36.2%)	151 (23.9%)	**22.875**	**0.000**
Sandwiches/pies/pizzas	451 (36.0%)	290 (46.7%)	161 (25.4%)	**61.547**	**0.000**
Cereals/nuts	198 (15.8%)	94 (15.1%)	104 (16.4%)	0.394	0.530
Fresh fruits/vegetables	467 (37.2%)	162 (26.1%)	305 (48.2%)	**65.485**	**0.000**
**Body weight stereotypes**					
Endorsement of thinness	496 (39.6%)	270 (43.5%)	226 (35.7%)	**7.926**	**0.005**
Anti-fat attitudes	762 (60.8%)	374 (60.2%)	388 (61.3%)	0.151	0.698
**BMI classification**				**71.630**	**0.000**
Underweight	53 (4.2%)	16 (2.6%)	37 (5.8%)		
Normal weight	826 (65.9%)	356 (57.3%)	470 (74.2%)		
Overweight	275 (21.9%)	171 (27.5%)	104 (16.4%)		
Obese	100 (8.0%)	78 (12.6%)	22 (3.5%)		
**Smoking behavior**					
Current smoker	360 (28.7%)	228 (36.7%)	132 (20.9%)	**38.536**	**0.000**
Exposure to maternal smoking	423 (33.7%)	255 (41.1%)	168 (26.5%)	**29.576**	**0.000**

^1^ Mean, standard deviation in parentheses. Statistically significant variables are marked in bold.

**Table 3:** Adolescents’ BMI estimates of the univariate ordered probit model.

Variables	BMI		
	Full Sample	Boys	Girls
Gender (girl)	−0.213 ***	**-**	**-**
	**(0.078)**		
Age	0.124 ***	0.140 ***	0.090 **
	**(0.029)**	**(0.041)**	**(0.043)**
Household size	−0.030	−0.083	0.044
	(0.039)	(0.053)	(0.061)
Mother’s educational level (high level)	−0.587 ***	−0.411 ***	−0.871 ***
	**(0.096)**	**(0.139)**	**(0.144)**
Father’s educational level (high level)	0.317 ***	0.220 *	0.434 ***
	**(0.085)**	**(0.115)**	**(0.133)**
**Snack choices**			
Dairy products	0.220 **	0.172	0.200
	**(0.089)**	(0.125)	(0.138)
Deep fried foods	0.618 ***	0.734 ***	0.272
	**(0.124)**	**(0.148)**	(0.253)
Confectionery	1.503 ***	1.352 ***	1.822 ***
	**(0.114)**	**(0.152)**	**(0.184)**
Sugar sweetened beverages	0.343 ***	0.273 **	0.507 ***
	**(0.086)**	**(0.121)**	**(0.131)**
Sandwiches/pies/pizzas	0.421 ***	0.551 ***	0.239 *
	**(0.084)**	**(0.114)**	**(0.132)**
Cereals/nuts	−0.567 ***	−0.864 ***	−0.222
	**(0.112)**	**(0.170)**	(0.157)
Fruits/vegetables	0.019	0.109 **	−0.093 *
	(0.033)	**(0.048)**	**(0.048)**
**Body weight stereotypes**			
Endorsement of thinness	0.270 ***	0.391 ***	0.255 **
	**(0.079)**	**(0.115)**	**(0.116)**
Anti-fat attitudes	−0.131 *	−0.373 ***	0.176
	**(0.079)**	**(0.114)**	(0.116)
**Smoking**	−0.404 ***	−0.275 *	−0.555 ***
	**(0.103)**	**(0.144)**	**(0.156)**
μ_1_	−0.081	−0.060	−0.205
μ_2_	2.917	3.057	2.875
μ_3_	4.472	4.718	4.421
**Log-Likelihood**	−808.868	−411.309	−371.395

Standard errors are given in parentheses. * *p* < 0.1, ** *p* < 0.05, *** *p* < 0.01. Statistically significant estimates are marked in bold.

**Table 4:** Adolescents’ smoking estimates—Treatment equation of the endogenous treatment effects model.

Variables	Smoking Behavior		
	Full Sample	Boys	Girls
Gender (girl)	−0.069	-	-
	(0.128)		
Age	0.413 ***	0.510 ***	0.366 ***
	**(0.047)**	**(0.066)**	**(0.072)**
Household size	0.077	−0.064	0.258 **
	(0.060)	(0.089)	**(0.102)**
Mother’s educational level (high level)	−0.327 **	−0.439 *	−0.271
	**(0.158)**	**(0.234)**	(0.230)
Father’s educational level (high level)	0.493 ***	0.576 ***	0.512 **
	**(0.140)**	**(0.201)**	**(0.224)**
**Snack choices**			
Dairy products	0.316 **	0.155	0.448 **
	**(0.143)**	(0.202)	**(0.226)**
Deep fried foods	0.134	0.263	−0.374
	(0.197)	(0.239)	(0.420)
Confectionery	0.404 **	0.506 **	0.308
	**(0.160)**	**(0.215)**	(0.251)
Sugar sweetened beverages	0.169	0.282	−0.068
	(0.133)	(0.189)	(0.204)
Sandwiches/pies/pizzas	0.551 ***	0.547 ***	0.634 ***
	**(0.133)**	**(0.183)**	**(0.205)**
Cereals/nuts	−0.219	0.225	−0.574 **
	(0.196)	(0.324)	**(0.292)**
Fruits/vegetables	0.022	−0.093	0.157 **
	(0.052)	(0.075)	**(0.076)**
**Body weight stereotypes**			
Endorsement of thinness	−0.515 ***	−0.914 ***	−0.211
	**(0.133)**	**(0.204)**	(0.193)
Anti-fat attitudes	−0.139	−0.066	−0.347 *
	(0.128)	(0.184)	**(0.195)**
**Exposure to maternal smoking (instrument)**	2.274 ***	2.367 ***	2.336 ***
	**(0.134)**	**(0.201)**	**(0.203)**
**Log-Likelihood Function**	−284.916	−141.926	−128.926

Standard errors are given in parentheses. * *p* < 0.1, ** *p* < 0.05, *** *p* < 0.01. Statistically significant estimates are marked in bold.

**Table 5:** Adolescents’ BMI estimates of the endogenous treatment effects model—Outcome equation.

Variables	BMI (Outcome Equation)		
	Full Sample	Boys	Girls
Gender (girl)	−0.201 ***	-	-
	**(0.077)**		
Age	0.077 **	0.053	0.080 *
	**(0.032)**	(0.045)	**(0.047)**
Household size	−0.027	−0.062	0.041
	(0.039)	(0.053)	(0.061)
Mother’s educational level (high level)	−0.590 ***	−0.446 ***	−0.868 ***
	**(0.096)**	**(0.137)**	**(0.144)**
Father’s educational level (high level)	0.298 ***	0.170	0.431 ***
	**(0.085)**	(0.115)	**(0.133)**
**Snack choices**			
Dairy products	0.220 **	0.207 *	0.195
	**(0.089)**	**(0.124)**	(0.138)
Deep fried foods	0.606 ***	0.669 ***	0.287
	**(0.124)**	**(0.149)**	(0.255)
Confectionery	1.387 ***	1.128 ***	1.796 ***
	**(0.120)**	**(0.160)**	**(0.190)**
Sugar sweetened beverages	0.332 ***	0.246 **	0.509 ***
	**(0.085)**	**(0.120)**	**(0.131)**
Sandwiches/pies/pizzas	0.386 ***	0.491 ***	0.229 *
	**(0.085)**	**(0.114)**	**(0.133)**
Cereals/nuts	−0.541 ***	−0.857 ***	−0.210
	**(0.111)**	**(0.167)**	(0.158)
Fruits/vegetables	0.017	0.104 **	−0.094 **
	(0.033)	**(0.048)**	**(0.048)**
**Body weight stereotypes**			
Endorsement of thinness	0.285 ***	0.436 ***	0.255 **
	**(0.079)**	**(0.114)**	**(0.116)**
Anti-fat attitudes	−0.127 *	−0.377 ***	0.179
	**(0.078)**	**(0.112)**	(0.116)
**Smoking**	−0.037	0.369 **	−0.469 **
	(0.142)	**(0.184)**	**(0.221)**
μ_1_	−0.676	−1.084	−0.356
μ_2_	2.287	1.936	2.720
μ_3_	3.840	3.571	4.270
*ρ*	−0.356 ***	−0.624 ***	−0.085
	**(0.092)**	**(0.099)**	(0.155)
**Log-Likelihood (both stages)**	−1087.504	−542.578	−500.171

Standard errors are given in parentheses. * *p* < 0.1, ** *p* < 0.05, *** *p* < 0.01. Statistically significant estimates are marked in bold.

**Table 6:** Marginal effects of smoking on adolescents’ BMI.

	Endogenous Treatment Effects Model with an Ordered Outcome		Univariate Ordered Probit Model	
	Marginal Treatment Effects *		Marginal Effects *	
BMI Categories	Boys	Girls	Boys	Girls
Underweight	**−0.018**	**0.060**	0.003	**0.045**
	(−0.117, −0.000)	(0.000, 0.176)	(−0.001, 0.006)	(0.010, 0.079)
Normal weight	−0.070	0.014	0.100	**0.055**
	(−0.145, 0.108)	(−0.172, 0.184)	(−0.001, 0.200)	(0.030, 0.080)
Overweight	0.044	−0.052	−0.090	**−0.095**
	(−0.117, 0.117)	(−0.143, 0.143)	(−0.178, 0.002)	(−0.140, −0.050)
Obese	**0.044**	**−0.022**	**−0.014**	**−0.005**
	(0.000, 0.146)	(−0.186, −0.000)	(−0.0289, −0.000)	(−0.008, −0.001)

* 95% Confidence intervals are reported in round brackets. Statistically significant estimates are marked in bold.

## Data Availability

The data selected for this study cannot be made publicly available. The ethics approval does not include consent for the availability of the datasets that support the conclusions of this article.
